# Short-Term Heart Rate Variability in Metabolic Syndrome: A Systematic Review and Meta-Analysis

**DOI:** 10.3390/jcm12186051

**Published:** 2023-09-19

**Authors:** Johan E. Ortiz-Guzmán, Sara Mollà-Casanova, Pilar Serra-Añó, Óscar J. Arias-Mutis, Conrado Calvo, Alexandra Bizy, Antonio Alberola, Francisco J. Chorro, Manuel Zarzoso

**Affiliations:** 1 Department of Physiology, Universitat de València, Av. Blasco Ibáñez 15, 46010 Valencia, Spain; jorguz@alumni.uv.es (J.E.O.-G.); conrado.calvo@uv.es (C.C.); antonio.alberola@uv.es (A.A.); 2 Department of Physiotherapy, Universitat de València, Street Gascó Oliag 5, 46010 Valencia, Spain; sara.molla@uv.es (S.M.-C.); pilar.serra@uv.es (P.S.-A.); 3 UBIC Research Group, Department of Physiotherapy, Universitat de València, Street Gascó Oliag 5, 46010 Valencia, Spain; 4 CEU Cardenal Herrera, Department of Biomedical Sciences, Street Lluís Vives 1, 46115 Valencia, Spain; oscarariasphd@gmail.com (Ó.J.A.-M.); alexandra.bizy@uchceu.es (A.B.); 5 Health Research Institute—Instituto de Investigación Sanitaria del Hospital Clínico Universitario de Valencia (INCLIVA) Valencia, Street Menéndez y Pelayo 4, 46010 Valencia, Spain; francisco.j.chorro@uv.es; 6 Centro de Investigación Biomédica en Red de Enfermedades Cardiovasculares (CIBER-CV) Madrid, Av. Monforte de Lemos, 3–5, Pavilion 11, Floor 0, 28029 Madrid, Spain; 7 CSIC-UPV, Instrumentation for Molecular Imaging Technologies Research Institute (I3M), Universitat Politècnica de València, 46022 Valencia, Spain; 8 Department of Medicine, Universitat de València, Av. Blasco Ibáñez 15, 46010 Valencia, Spain

**Keywords:** heart rate variability, short-term recording, metabolic syndrome, cardiac autonomic dysfunction

## Abstract

Background: Our aim was to determine the differences in short-term heart rate variability (HRV) between patients with metabolic syndrome (MS) and healthy controls. Methods: We searched electronic databases for primary works with short-term HRV recordings (≤30 min) that made comparisons between individuals with MS versus healthy controls. This systematic review and meta-analysis (MA) was performed according to PRISMA guidelines and registered at PROSPERO (CRD42022358975). Results: Twenty-eight articles were included in the qualitative synthesis and nineteen met the criteria for the MA. Patients with MS showed decreased SDNN (−0.36 [−0.44, −0.28], *p* < 0.001), rMSSD (−7.59 [−9.98, −5.19], *p* < 0.001), HF (−0.36 [−0.51, −0.20], *p* < 0.00001) and LF (−0.24 [−0.38, −0.1], *p* = 0.001). In subsequent subanalyses, we found a decrease in SDNN (−0.99 (−1.45, −0.52], *p* < 0.001), rMSSD (−10.18 [−16.85, −3.52], *p* < 0.01) and HF (−1.04 [−1.97, −0.1] *p* < 0.05) in women. In men, only LF showed a significant lower value (−0.26 [−0.5, −0.02], *p* < 0.05). We could not perform MA for non-linear variables. Conclusions: Patients with MS showed changes in time-domain analyses, with lower values in SDNN and rMSSD. Regarding frequency-domain analyses, MS patients showed a decrease in HF and LF When sex was used as a grouping variable, the MA was only possible in one of both sexes (men or women) in rMSSD and LF/HF. Lastly, when data for both men and women were available, subanalyses showed a different behavior compared to mixed analyses for SDNN, HF and LF, which might point towards a different impact of MS in men and women.

## 1. Introduction

Metabolic syndrome (MS) consists of a diverse combination of cardiometabolic risk factors and some associated conditions, such as abdominal obesity, decreased HDL and increased LDL cholesterol levels, elevated triglycerides, impaired glucose tolerance, and hypertension, that predispose to high risk of both cardiovascular disease and type 2 diabetes [1,2]. It is estimated that between 20% and 25% of the adult population worldwide has MS [3], a prevalence that tends to increase due to the current lifestyle habits, especially in western societies. The exposure to artificial light, constant availability of processed and ultra-processed food, and excess of stressful stimuli in daily or work environments, among other conditions, favor the development of cardio-metabolic risk factors and related comorbidities, like cardiovascular diseases, type 2 diabetes, obesity and cancer [4,5,6].

Regarding non-communicable chronic diseases (NCDs), scientific evidence links their appearance and progression to a state of dysregulation of the autonomic nervous system (ANS), in which a predominance of sympathetic tone is observed [7,8]. For some authors, the appearance of an alteration in the regulation of the sympathetic nervous system could be a red flag and a primary risk factor for the development of MS, thus the development of therapeutic actions focused on autonomic monitoring and control could help to detect the severity of MS, as well as attenuating its adverse outcomes [9].

Therefore, the evaluation of ANS function could be a very useful tool when diagnosing or monitoring patients with MS. Indeed, the study of heartbeat fluctuations through heart rate variability (HRV) is an interesting and non-invasive measurement method which allows to observe and analyze the behavior of cardiac autonomic balance [10]. Long (24 h), short (5–15 min) and ultra-short (10–20 s) term recordings have been used to assess changes in HRV in MS patients in order to evaluate the degree of autonomic dysfunction, which predisposes to cardiac arrhythmic events and sudden cardiac death [11]. Indeed, several descriptive and randomized controlled trials that analyzed short-term HRV, have identified modifications in some of its components, but in many cases, the results are not consistent, as shown by recent studies [12]. Furthermore, despite the fact that severity and the number of components of MS seem to be associated with changes in HRV [13], to date no previous study has quantitatively synthesized (i.e., meta-analysis) the findings of published research that describe the disfunction in the ANS presented in MS, using short-term HRV.

Hence, we undertook this systematic review and meta-analysis in order to determine the differences in short-term heart rate variability (HRV) between patients with and without MS, which would enable us to characterize the cardiac autonomic dysfunction induced by this pathological condition and the most frequently reported explanatory variables.

## 2. Materials and Methods

### 2.1. Search Strategy

The International Prospective Register of Systematic Reviews (PROSPERO, https://www.crd.york.ac.uk/prospero/ (accessed on 19 October 2022)) was used to register the study (CRD42022358975). Moreover, the PRISMA guidelines (Preferred Reporting Items for Systematic Reviews and Meta-analyses) [14] was used to conduct it. The search was made in the specialized databases of the US National Library of Medicine (PubMed), the Web of Science and Scopus, and was delimited in the review topic by keywords linked to specific MESH terms. The terms used in these database were “metabolic syndrome” linked to “heart rate variability”, “cardiac autonomic control”, “cardiac autonomic function”, or “cardiac autonomic modulation”. The boolean characters AND and OR were used to construct the search equations, and the exploration was limited to the title and abstract fields (Table 1). With these search equations, we searched for original works between 25 June 2022, and 1 September 2022.

### 2.2. Study Inclusion/Exclusion Criteria

The inclusion criteria for this systematic review and metanalysis were: (i) original works; (ii) performed in humans; (iii) with HRV recordings shorter than 30 min (between 2 and 30 min) (iv) with comparisons data between people with diagnosed with MS (MS+) versus healthy (i.e., people without MS) as a control group (MS−); (v) that analyses time or frequency domains or non-linear variables; and (vi) written in English. A detailed description of the variables can be found in Supplementary Tables S1–S3.

Exclusion criteria included (i) systematic reviews of the literature and/or meta-analyses, (ii) bibliographic reviews, (iii) letters to the editor or (iv) conference communications.

### 2.3. Quality Assessment

To assess the methodological quality of the studies, the recommendations made by Law et al. [15,16] for the review of quantitative articles were used. The scale includes 16 items to assess each article, and the result is expressed as a percentage, which is calculated by adding the total number of items contained and dividing this result by 16. In accordance with this, the articles with a less or equal score than 50% were classified as “low methodological quality”, between 51 and 75% as “good methodological quality”; and a score higher than 75% was defined as an “excellent methodological quality” [16].

### 2.4. Data Extraction

The main information of each work was collected in a Microsoft Excel (2019) table, including general information of the study (title, authors, journal and year of publication, objective), subjects’ characteristics (sample size by groups, sex, age, weight, height, Body Mass Index-BMI-), HRV recordings procedures (recording time and hour, body position, ventilation control, previous fasting, time and frequency and non-linear variables, criteria for MS diagnostic, and key outcomes.

A total of 28 studies were included in the qualitative syntesis (i.e., systematic review). Then, an accurate qualitative synthesis was performed. Methodological and clinical diversity (i.e., heterogeneity) was assessed in order to determine if a meta-analysis (MA) was appropriate. Therefore, the I^2^; statistical test was used following the recommendations of Cochrane Handbook [17]: non-important heterogeneity was ranged between 0–40%; moderate heterogeneity was ranged between 30%–60%; substantial heterogeneity was ranged between 50–90%; and considerable heterogeneity was ranged between 75–100% [17]. To detect potential differences in the methodology of included studies, high heterogeneity was taken into account. Moreover, to reduce risk of bias and the heterogeneity, the studies with less than 5 min or 250 intervals of recording were excluded from the quantitative synthesis (i.e., MA) [11].

The Review Manager software 5.4 for Windows (RevMan Version 5.4, The Nordic Cochrane Center, The Cochrane Collaboration, Copenhagen, Denmark) was used to perform the MA and to analyze the differences between groups. It was conducted when 3 or more articles assessed and measured the same outcome. Before pooling the data, comparisons were grouped as MS+ versus MS−, and Cohen’s d and the 95% Confidence Interval (CI) was used to report the differences between these two groups. Standard mean difference (SMD) was used when different outcome measures were combined; while mean difference (MD) was used when different outcome measures were not combined.

Finally, subgroup analyses were carried out taking into account the factor “sex”. This analysis was only performed when three or more studies included separate data for both men and women.

## 3. Results

### 3.1. Identification of Studies

**Figure 1:**
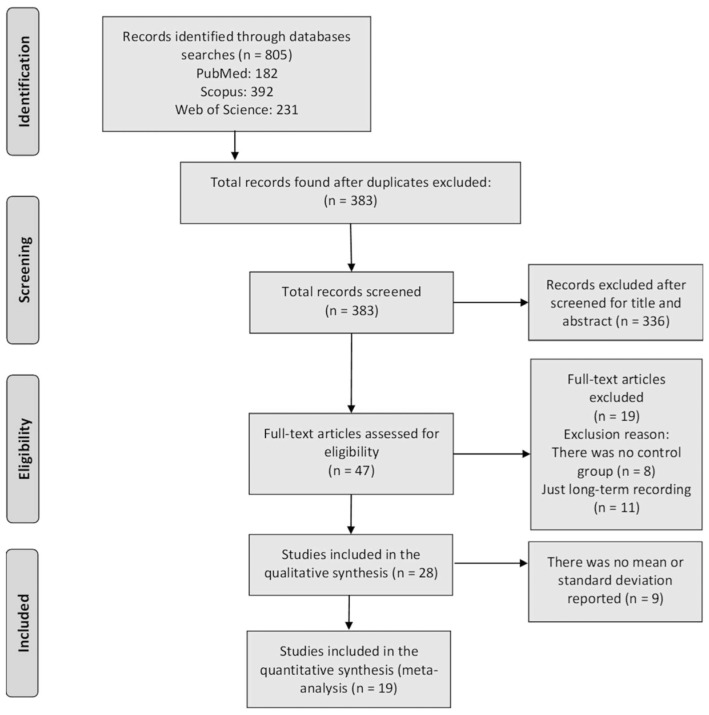
Flowchart of article selection.

In the initial review, 805 articles were identified (182 in PUBMED, 392 in Scopus and 231 in Web of Science). The duplicated studies between selected databases were excluded (422), title and abstract of the remained 383 records were reviewed and a total of 336 additional studies were excluded (201 for not measuring HRV, 61 for analyzing only 1 component of MS, 48 for not being original articles and 26 for being studies in animal models). Subsequently, 47 full-text papers were retrieved, which were reviewed to ensure that they met the inclusion and exclusion criteria. Of these, 8 were excluded from the final analysis for not making comparisons between people with MS+ vs. MS−, and 11 for just reporting long-term recordings. No study was considered as “low methodological quality”, thus 28 articles were included in the final qualitative synthesis (Figure 1). Finally, 9 more articles were excluded from the quantitative meta-analysis (MA), as they did not report mean and/or standard deviations. Thus, the MA was carried out with 19 articles (Figure 1).

### 3.2. Quality Assessment

The methodological evaluation showed 82% (23) of the articles valued as “excellent methodological quality”, 18% (5) as “good methodological quality” and no reviewed study was scored as “low methodological quality”. The mean scored of the methodological quality of the reviewed article was 85% (“excellent methodological quality”).

### 3.3. Study and Patient Characteristics

The main studies’ characteristics reviewed are shown in Table 2. We found 1 study published before the year 2000, 9 between 2002 and 2010, and 18 between 2012 and 2020. The population under investigations originated from: North America (n = 4), including 3 from the United States [18,19,20] and 1 from Canada [21]; South America (n = 5), all of them from Brazil [9,10,22,23,24]; Europe (n = 7), finding 1 from England [25], 3 from Finland [26,27,28], 1 from France [29], 1 from Portugal [30] and 1 from Serbia [31]; and Asia (n = 12), where 4 were from Taiwan [32,33,34,35], 3 from South Korea [36,37,38], 2 from China [39,40], 2 from India [41,42], and 1 from Japan [43].

Regarding sex, 3 studies included only men [19,25,38], 3 studies included only women [9,22,23] and 22 studies were carried out in both sexes [10,18,20,21,26,27,28,29,30,31,32,33,34,35,36,37,39,40,41,42,43,44]. With respect to age, 5 papers included people between 18 and 40 years old [10,22,26,30,41], 16 papers evaluated the population over 40 years old [9,18,19,20,23,25,28,29,31,32,35,38,39,40,42,44], 6 more included populations between 18 and 79 years old [21,33,34,36,37,43] and 1 study was done in children between 6 and 8 years old [27]. The 68% (12,499) of all individuals included in this review (18,440) were people with MS.

Following the criteria described for the quantitative analysis, the variables R-R, pNN50, NN50, RRtri, TINN and all non-linear variables were not analyzed quantitatively (highlighted in bold in Table 2). The other linear variables, both in the time domain and frequency domain, were included in the quantitative analysis (i.e., SDNN, rMSSD, HF, LF, LF/HF). According to the recommendations from the Task Force of The European Society of Cardiology and The North American Society of Pacing and Electrophysiology [11], VLF and TP were not analyzed either, since their analysis is not recommended in short-term recordings given that their physiological explanation is much less defined and the existence of a specific physiological process attributable to these heart period changes might even be questioned. All the articles included in the MA analyzed the HRV from cardiac recordings.

### 3.4. Time Domain Analysis Outcomes

In total, 17 of the 28 studies included in this review analyzed time domain variables. The main results of the qualitative analyses are provided in Table 3.

In the case of the R-R interval, lower values were reported in men older than 40 years with MS [25] and in women between 20 and 40 years with MS+ [22]. Other studies also reported significantly lower values in R-R when analyzing data from mixed groups [10,41,42,44]. Stuckey et al. reported a decrease in the R-R interval when performing the analysis only in women but not when combining men and women in the same group [21]. Regarding pNN50, two studies report lower values in men and women with MS+ [41,42], one study only in women [23] and another study did not find differences between groups [10].

**Figure 2:**
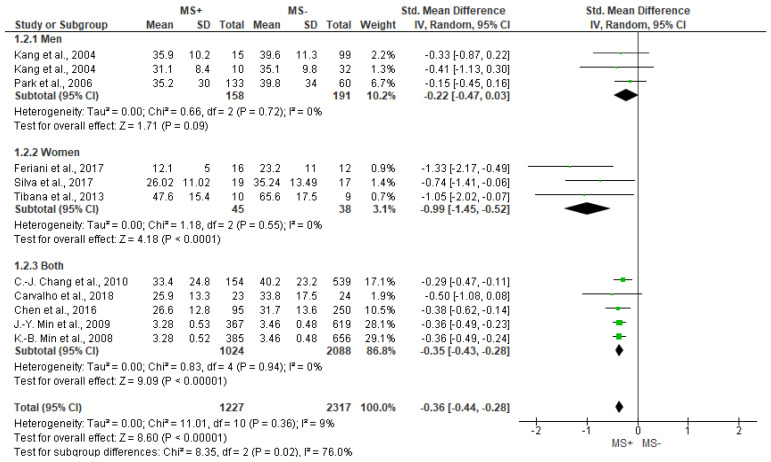
Forest plot showing the SDNN between metabolic syndrome (MS+) versus control (MS−).

The SDNN outcome is the most studied by the different works reviewed. Figure 2 shows significant differences between groups for SDNN outcome, being lower in people with MS+ (SMD = −0.36 [95%IC = −0.44, −0.28], *p* < 0.001), with low heterogeneity between reports (I^2^ = 9%). In addition, sub analyses showed significant differences between groups in women (−0.99 [−1.45, −0.52], *p* < 0.001)*,* but not in men (*p* = 0.09).

**Figure 3:**
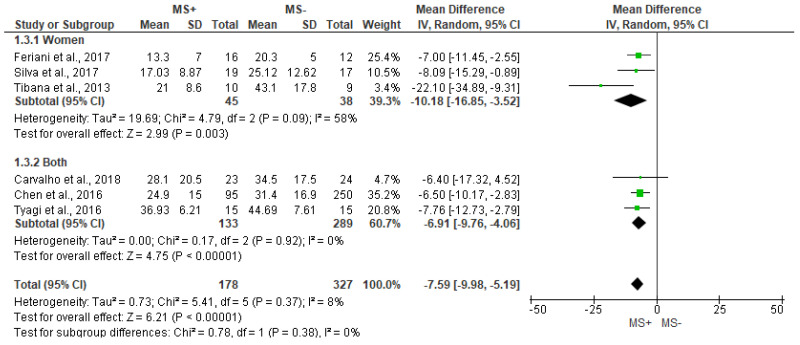
Forest plot showing the rMSSD between metabolic syndrome (MS+) versus control (MS−).

Significant differences between groups were found for rMSSD (MD = −7.59 [95%IC = −9.98, −5.19], *p* < 0.001), with small heterogeneity between studies (I^2^ = 8%, Figure 3). In the same way, women also showed lower values of rMSSD in the MS+ group when compared with MS− (−10.18 [−16.85, −3.52], *p* = 0.003), while differences between groups could not be assessed in men sub analysis, due to lack of data.

### 3.5. Frequency Domain Analysis Outcomes

For the spectral variables of the HRV, 25 studies carried out frequency domain analyses. A summary of the main results about its qualitative is showed in Table 4.

**Figure 4:**
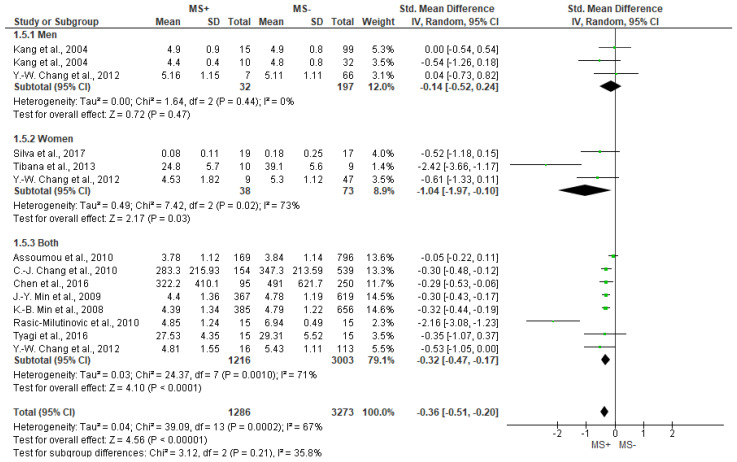
Forest plot showing the HF between metabolic syndrome (MS+) versus control (MS−).

Regarding HF outcome, and with substantial heterogeneity, Figure 4 shows significant differences between groups (SMD = −0.36 [−0.51, −0.20], *p* < 0.001), showing lower values for the MS+ group (men and women together) (−0.32 [−0.47, −0.17], *p* < 0.001) and women only (−1.04 [−1.97, −0.10], *p* = 0.03), but not for men (*p* = 0.47).

**Figure 5:**
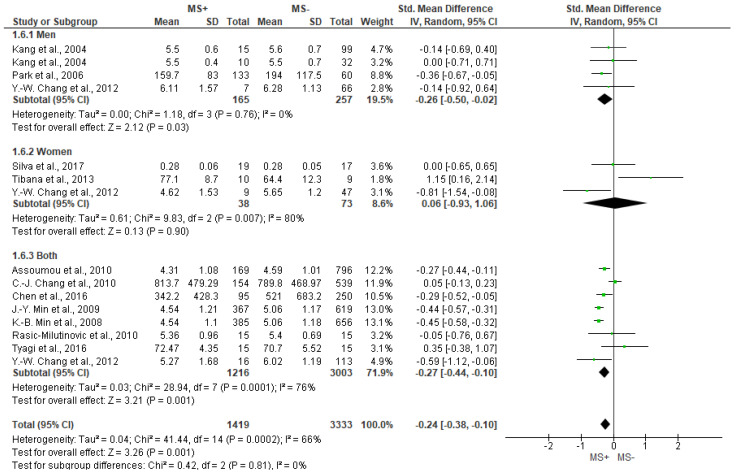
Forest plot showing the LF between metabolic syndrome (MS+) versus control (MS−).

Figure 5 depicts the different analyses of LF. Significant lower values of LF for MS+ group compared to MS− were found (SMD = −0.24 [−0.38, −0.1], *p* = 0.001), even though the results show substantial heterogeneity (I^2^ = 66%). Regarding subgroup analyses, mixed (both men and women) (−0.27 [−0.44, −0.1], *p* = 0.001) and men groups also showed significantly lower values of LF for MS+ group (−0.26 [−0.50, −0.02], *p* = 0.03) compared with MS−, while women did not show significant differences between groups (*p* = 0.90).

**Figure 6:**
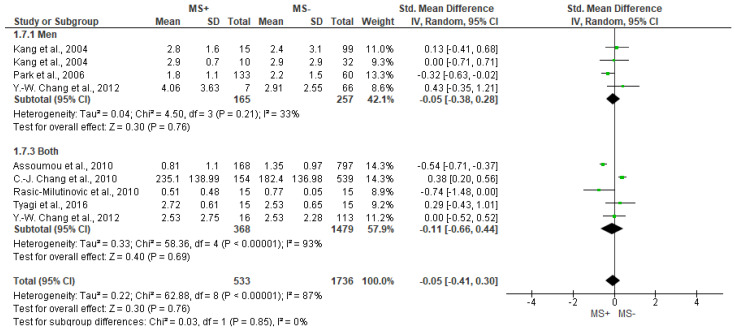
Forest plot showing the LF/HF between metabolic syndrome (MS+) versus control (MS−).

Regarding the LF/HF outcome, we found high heterogeneity (I^2^ = 87%) between studies and no significant differences (*p* = 0.76) were found between groups in both the general analysis and subanalyses (Figure 6).

### 3.6. Non-Linear Analysis Outcomes

Regarding the non-linear analysis, only 3 studies included these variables (Table 5). Stuckey et al. found no significant differences for SD1 of the Poincare Plot, for men or women, but they did find a significant increase for SD2 in MS+ women compared to MS− women [21]. No significant differences were found in α1 (detrended fluctuations analysis) or approximate entropy (ApEn) [21]. Silva et al. analyzed the records of 19 MS+ women and compared them to the records of 17 sex-match MS−, but they did not report differences in entropy (Shannon entropy, ShanEn) [9]. Finally, Carvalho et al. found a significant decrease for SD1 in young men and women, but not in SD2 and α1 of the Poincare analysis, or ShanEn [10].

## 4. Discussion

We conducted this systematic review and MA to determine the difference in short-term heart rate variability (HRV) between patients with metabolic syndrome (MS) and healthy controls. A total of 28 moderate-high quality studies were reviewed, 19 of which were included in the MA.

The main findings were: (1) patients with MS showed changes in short-term HRV in the time-domain analyses, having a decrease of all the parameters included in the MA: SDNN and rMSSD; (2) regarding the frequency-domain analyses, MS group reported a decrease in HF and LF, with the exception of the LF/HF relationship, which was not modified; (3) when sex was taken into account, the MA was only possible in one of both sexes (men or women) in rMSSD and LF/HF; (4) when data for both men and women were available, sex subanalyses showed a different behavior that the mixed analyses for SDNN, HF and LF, which might point towards a different impact of MS in men and women, (5) we could not perform the MA in non-linear parameters, RR and pNN50 due to the lack of studies and data availability.

It has been suggested that the SDNN is an indicator of the global behavior of the HRV [9,19,45] which being dependent on the analysed signal length, has shown to correlate well with the TP of the spectral analysis [11]. The MA showed that SDNN is decreased in patients with MS, which was previously described by most studies [10,18,32,34,36,37], thus clearly altering cardiac autonomic control in patients with MS. This suggests overall lower HRV, and therefore, reduced parasympathetic cardiac control in MS patients.

The other variable of the time-domain analyses reviewed in this MA is the rMSSD, directly linked to short-term HRV components [46], which is corelated to HF of the frequency-domain [11] and provides information on parasympathetic activity [9,45]. We found a decrease in rMSSD in people with MS, in both the mixed group and the subgroup of women. For Carvalho et al. [10], MS in young adults (<40 years) causes a decrease in parasympathetic modulation, which is also reported by Chen et al. [34] and Tyagi et al. [41] in those older than 40 years. It has been reported that in MS, C-reactive protein levels are increased because of a chronic condition of low-grade inflammation, which could cause the ANS dysregulation [34,47], thus increasing sympathetic activity and decreasing parasympathetic activity. For some authors, the deterioration of cardiac parasympathetic modulation is the main cause that would explain the alteration in autonomic control in MS, also suggesting that this worsening is closely related to fasting glycemia levels [9], states of insulin resistance [48] and type 2 diabetes [49].

In the frequency-domain analyses, the decrease in HF found in the MA reinforces the hypothesis that MS favors a lower parasympathetic activity [11,45]. HF presents significantly lower values in people with MS, both in the analysis of the data for men and women together (mixed group), and for the subgroup of women. On the other hand, the LF component of the spectral analysis, which has been suggested to reflect the sympathetic behavior exclusively by some studies [10,50], and as a parameter that could be modulated by both the sympathetic and parasympathetic branches of the ANS by others [9,45,51,52,53], decreased in the mixed group. LF components have been related in part to baroreflex control of HR to maintain BP homeostasis. Some studies have identified a relation between reduced neural baroreflex pathway and baroreceptor resetting with MS subjects and those at risk of high BP [54]. A weaker baroreflex response results in less efficient BP maintenance and lower HRV. The explanation of the underlying mechanisms could be difficult to interpret due to the controversy about its main modulating mechanism, likely multifactorial and non-linear, but it is important to emphasize that the LF component is decreased with MS, which indicates a lower HRV. Finally, since both LF and HF decreased, the MA showed no significant changes in the ratio LF/HF. This might be aligned with previous doubts by some authors casting that the LF/HF ratio should not be considered as a strong index to analyse the balance between the sympathetic and parasympathetic activities [55] since LF might itself be biased as a solid sympathetic indicator.

Regarding the analysis by sex, MS+ women group showed significantly lower values of SDNN compared with those in the MS− group, while men sub analysis did not show significant differences. Some authors have proposed that this behavior could be explained by physiological stress that, in the case of women, is exacerbated even at early ages, due to a greater systemic response to inflammatory processes [45,56]. In addition, LF was decreased only in men and, due to the important role that central obesity plays in the etiology of MS [26], it is important to highlight that men included in the studies reviewed in this MA showed high rates of central obesity, which favours chronic exposure to oxidative stress and increased risk of autonomic dysfunction, as has been reported [19,26]. However, LF was not modified in the group of women which could be explained, at least in part, by the fact that in premenopausal women the accumulation of abdominal fat is less common than in men at the same ages. Since central obesity is considered a key factor in the etiology of MS [26,33], the age of women who were evaluated in the studies included in the MA could explain the results. On the other hand, SDNN [19,38] and HF [33,38] did not reach statistical significance in men, even though the reviewed studies agree that these values showed a tendency to be lower, suggesting that parasympathetic modulation could be decreased also in men with MS.

HRV temporal series are non-stationary and non-linear in nature, likely due to non-linear interaction amongst different cardiovascular regulatory mechanisms. The non-linear analysis has been proposed to be a tool with high value for the analysis of complex systems and the predictability of a time series, which results from the complexity of the mechanisms that regulate HRV, analyzing the fractal behavior of the signal (detrended fluctuations analysis) and the complexity or regularity in the data series (entropy) [21]. The works published to date, however, do not allow a MA of these variables. Nevertheless, the available evidence points towards a decrease of SD1 in both men and women with MS [10], while SD2 seems to increase in women with MS [21]. SD1 has been associated directly to parasympathetic activity [57] and correlating well with SDNN and rMSSD, while SD2, has been reported to be related to both sympathetic and parasympathetic activity. SD2 has also been inversely related to sympathetic activity in sedentary and non-sedentary subjects [58]. On the other hand, DFA and entropy do not show significant changes in MS group compared with controls [9,10,21]. However, it is important to emphasize that, as indicated above, these are findings reported by a small sample of studies, so future works that analyze the non-linear behavior of HRV would be necessary to reach conclusive analyses, even though some authors question the physiological significance of using the non-linear analysis for short-term recordings [59].

Among cardiovascular complications, cardiac autonomic dysfunction is one of the most important, and it has been associated with a significant increase in morbidity and mortality independent of other risk factors [60,61]. Indeed, it has been reported that reduced HRV is a marker of autonomic dysfunction, and it is already evident in patients with MS, even before the development of type-2 diabetes [62]. Thus, early detection of autonomic dysfunction in prediabetic patients using HRV would be as important as the screening for MS, since the progression of cardiovascular denervation is partly reversible or can be delayed in the early stages of the disease [63,64].

## 5. Limitations

One of the main limitations of the MA is the high degree of heterogeneity presented in some of the parameters studied. Likewise, due to the low number of studies included in certain analyses, it was not possible to analyze the behavior of short-term HRV by sex in some variables, especially in the case of women. In addition, more studies are required to be able to develop quantitative analyzes about the non-linear variables of HRV, even though its clinical significance and utility in short-term recordings is questionable. On the other hand, and taking into account that the behavior of the HRV is largely conditioned by factors such as the body position in which the recordings are made, electrode position and/or recording method, the time of day and circadian rhythms, whether or not there was fasting prior to the recordings, the use of drugs and respiratory rate should be clearly reported in studies measuring HRV. Such factors may have indeed greater impact on short-term recordings than long-term recordings. Several recording methods have been used in the analysed studies, such as ECG measurements, RR recordings with digital heart rate bands, and pulse-wave recordings. However, in order to reduce variability and heterogeneity, only HRV analyses derived from ECG recordings were included in the MA. In addition, the recent development and use of wearable devices and phone applications might be useful for the study of HRV, specially for long-term recordings, as well as for obtaining self-reported assessments in the patient’s natural environment, but a meticulous process of validation is needed for their use in scientific research. In this line, efforts should be made to standardize the recording protocols, which in turn would reduce variability in the measurements and would improve data quality.

## 6. Conclusions

In conclusion, we found that patients with MS had differences in short-term HRV in the time-domain analyses, showing a decrease of SDNN and rMSSD. Regarding the frequency-domain analyses, patients with MS showed a decrease in HF and LF. When sex was taken into account, the MA was only possible in one of both sexes (men or women) in rMSSD and LF/HF. Lastly, when data for both men and women were available, sex subanalyses showed a different behavior than the mixed analyses for SDNN, HF and LF, which might point towards a different impact of MS in men and women. Regarding the analysis of non-linear variables (entropy, DFA, Poincare Plot), the results are not conclusive, due to the low number of datasets found for analysis.

## Figures and Tables

**Table 1:** Search strategy.

Database	Search Equation
PubMed	((“heart rate variability” [Title/Abstract] or “autonomic control” [Title/Abstract] or “HRV” [Title/Abstract] or “cardiac autonomic control” [Title/Abstract] or “cardiac autonomic function” [Title/Abstract] or “cardiac autonomic modulation” [Title/Abstract]) AND (“metabolic syndrome” [Title/Abstract]))
Web of Science	(“heart rate variability” or “autonomic control” or “HRV” or “cardiac autonomic control” or “cardiac autonomic function” or “cardiac autonomic modulation”) and (“metabolic syndrome”)
Scopus	(TITLE-ABS-KEY (“metabolic syndrome”) and TITLE-ABS-KEY (“heart rate variability”) or TITLE-ABS-KEY (“autonomic control”) or TITLE-ABS-KEY (“HRV”) or TITLE-ABS-KEY (“cardiac autonomic control”) or TITLE-ABS-KEY (“cardiac autonomic function”) or TITLE-ABS-KEY (“cardiac autonomic modulation”))

**Table 2:** Summary of the studies.

Reference	Methodological Evaluation (%)	n	Age (Years)	Gender	MS Definition	Recording Characteristics		Analyzed HRV Variables			
						Recording Time (min)	Body Position	Time	Frequency	Spectral Methods	Non-Linear
Liao et al., 1998 [18]	81%	2359	45–64	Both	HTA, DM-2, dislipidemia	2	Supine	SDNN	HF, LF, LF/HF	FFT	No
Brunner et al., 2002 [25]	63%	183	45–63	Men	NCEP-ATP III	5	Supine	SDNN	TP, LF, HF	Blackman-Tukey	No
Kang et al., 2004 [38]	69%	156	41–55	Men	≥3 risk factors	5	Sitting	SDNN, rMSSD	HF, LF, LF/HF	NR	No
Park et al., 2006 [19]	88%	413	64–79	Men	NCEP-ATP III	7	Sitting	SDNN	HF, LF, LF/HF	FFT	No
K.-B. Min et al., 2008 [36]	88%	1041	20–87	Both	NCEP-ATP III, IDF	5	Sitting	SDNN	LF, HF	NR	No
J.-Y. Min et al., 2009 [37]	69%	986	20–87	Both	NCEP-ATP III	5	Sitting	SDNN	LF, HF	FFT	No
Koskinen et al., 2009 [26]	88%	1889	24–39	Both	NCEP-ATP III, IDF, EGIR	3	Supine	No	LH, HF, TP, LF/HF	FFT	No
Assoumou et al., 2010 [29]	94%	1010	64–66	Both	NCEP-ATP III	5	Supine	No	TP, HF, LF, VLF, ULF, LF/HF	FFT	No
C.-J. Chang et al., 2010 [32]	75%	1289	36–48	Both	NCEP-ATP III	5	Supine	SDNN	HF, LF, LF/HF	FFT	No
Rasic-Milutinovic et al., 2010 [31]	88%	47	45–65	Both	NCEP ATP III	NR	NR	SDNN rMSSD	HF, LF, LF/HF, TP, VLF	FFT	No
Y.-W. Chang et al., 2012 [33]	88%	129	19–62	Both	NCEP-ATP III	5	Supine	No	LH, HF, TP, LF/HF	FFT	No
Soares-Miranda et al., 2012 [30]	81%	163	19–21	Both	N/A	5	Supine	rMSSD, SDNN, NN50, **pNN50**	HF, LF/HF	FFT	SD1, SD2
Tibana et al., 2013 [22]	88%	19	30–40	Women	NCEP-ATP III	5	NR	**R-R**, SDNN, rMSSD	HF, LF, LF/HF	FFT	No
Li et al., 2013 [39]	94%	2119	50–70	Both	NCEP-ATP III	15	Supine	No	LH, HF, TP, LF/HF	NR	No
Stuckey et al., 2015 [21]	88%	220	23–70	Both	NCEP-ATP III	5	Supine	SDNN, rMSSD	LF, HF	FFT	SD1, SD2, α1, Aprox. Entropy
Chen et al., 2016 [34]	88%	345	20–65	Both	¤	5	NR	SDNN, rMSSD	VLF, LF, HF, TP	FFT	No
Tyagi et al., 2016 [41]	56%	30	40–50	Both	IDF	5	NR	rMSSD, **pNN50**, R-R	HF, LF, LF/HF	FFT	No
Y.-M. Chang et al., 2016 [35]	88%	175	50–80	Both	IDF	5	Supine	No	LF, HF, LF/HF, TP, VLF	FFT	No
Silva et al., 2017 [9]	94%	36	40–50	Women	§	12	Sitting	SDNN, rMSSD	HF, LF, LF/HF	FFT	Shannon Entropy
Feriani et al., 2017 [23]	94%	28	65–75	Women	NCEP-ATP III	20	NR	SDNN, rMSSD, **pNN50**	HF, LF, LF/HF	FFT	No
Saito et al., 2017 [43]	94%	2016	30–79	Both	≥3 risk factors	5	NR	SDNN, rMSSD	HF, LF, LF/HF	NR	No
Pennathur et al., 2017 [20]	94%	50	40–60	Both	NCEP-ATP III	5	Supine	No	HF, LF, LF/HF	Wavelet transform	No
Guo et al., 2018 [40]	100%	2476	45–70	Both	NCEP-ATP III	5	Sitting	No	LF, HF, LF/HF, VLF, TP	NR	No
Carvalho et al., 2018 [10]	88%	66	30–40	Both	NCEP-ATP III	300 consecutives R-R intervals	Supine	**R-R**, rMSSD, **pNN50**, **RRtri**, **TINN**	No	N/A	SD1, SD2, α1, Shannon Entropy
MacAgnan et al., 2019 [44]	88%	14	40–60	Both	NCEP ATP III	250–350 consecutives R-R intervals	Supine	R-R	HF, LF, LF/HF	Autoregressive algorithm	No
Kangas et al., 2019 [28]	88%	572	40–60	Both	IC	5	Supine	No	TP, LF, HF, LF/HF	FFT	No
Leppanen et al., 2020 [27]	94%	443	6–8	Both	NR	5	Supine	**R-R**, rMSSD	HF, LF, LF/HF	NR	No
Endukuru et al., 2020 [42]	94%	176	40–55	Both	NCEP ATP III	5	Supine	**R-R**, SDNN, **pNN50**, **NN50**, rMSSD	LF, HF, LF/HF, VLF, TP	NR	No

NCEP-ATP III: National Cholesterol Education Program’s Adult Treatment Panel III. IDF: International Diabetes Federation. EGIR: European Group for the Study of Insulin Resistance. HTA: Arterial hypertension. DM-2: Type 2 Diabetes Mellitus. ¤ The country-specific definition applied by Taiwan’s Ministry of Healthand Welfare. § Waist circumference (≥80 cm) and the presence of at least two criteria. IC: Consensus definition from several national and international organizations. min: minutes. FFT: Fast Fourier transformation. NR: not reported. N/A: Not Applicable.

**Table 3:** Reported changes in time domain analyses (short-term HRV) in MS.

Reference	SDNN	rMSSD	R-R	pNN50
Liao et al., 1998 [18]	↓			
Brunner et al., 2002 [25]	↓		↓	
Kang et al., 2004 [38]	↓	=		
Park et al., 2006 [19]	=			
K.-B. Min et al., 2008 [36]	↓			
J.-Y. Min et al., 2009 [37]	↓			
C.-J. Chang et al., 2010 [32]	↓			
Tibana et al., 2013 [22]	↓	↓	↓	
Stuckey et al., 2015 [21]	↓^w^	=	↓^w^	
Chen et al., 2016 [34]	↓	↓		
Tyagi et al., 2016 [41]		↓	↓	↓
Silva et al., 2017 [9]	↓	↓		
Feriani et al., 2017 [23]	↓	↓		↓
Saito et al., 2017 [43]	=	↓		
Carvalho et al., 2018 [10]	=	↓	↓	=
MacAgnan et al., 2019 [44]			↓	
Endukuru et al., 2020 [42]	↓	↓	↓	↓

w: only in women; =: without change; ↓: lower values in MS group.

**Table 4:** Reported changes in frequency domain analyses (short-term HRV) in MS.

Reference	HF	LF	LF/HF
Liao et al., 1998 [18]	↓	↓	=
Brunner et al., 2002 [25]	↓	↓	
Kang et al., 2004 [38]	=	=	=
Park et al., 2006 [19]	=	=	=
K.-B. Min et al., 2008 [36]	↓	↓	
J.-Y. Min et al., 2009 [37]	↓	↓	
Koskinen et al., 2009 [26]	↓	↓	↑^w^
Assoumou et al., 2010 [29]	=	↓	↓
C.-J. Chang et al., 2010 [32]	↓	=	↑
Rasic-Milutinovic et al., 2010 [31]	↓		
Y.-W. Chang et al., 2012 [33]	=	=	=
Tibana et al., 2013 [22]	↓	↑	↑
Li et al., 2013 [39]	↓	↓	↓
Stuckey et al., 2015 [21]	=	↑^w^	=
Chen et al., 2016 [34]	↓	↓	
Tyagi et al., 2016 [41]	↓	↑	↑
Y.-M. Chang et al., 2016 [35]	=	=	=
Silva et al., 2017 [9]	↓	=	↑
Feriani et al., 2017 [23]	↓	↑	↑
Saito et al., 2017 [43]	↓	=	↑
Pennathur et al., 2017 [20]	=	=	↑
Guo et al., 2018 [40]	↓	↓	=
MacAgnan et al., 2019 [44]	↓	↑	↑
Kangas et al., 2019 [28]	↓	↓^m^	=
Endukuru et al., 2020 [42]	↓	↓	↑

^m^: only in men; w: only in women; =: without change; ↓: lower values in MS group; ↑: upper values in MS group.

**Table 5:** Reported changes in non-linear analyses (short-term HRV) in MS.

Reference	SD1	SD2	α1	ApEn	ShanEn
Stuckey et al., 2015 [21]	=	↑^w^	=	=	
Silva et al., 2017 [9]					=
Carvalho et al., 2018 [10]	↓	=	=		=

w: only in women; =: without change.

## Data Availability

All data generated or analyzed during this study are included in the published studies and their supplementary information files.

## Supplementary Materials

The following supporting information can be downloaded at: https://www.mdpi.com/article/10.3390/jcm12186051/s1, Table S1: Description of main time-domain HRV measurements reviewed; Table S2: Description of main frequency-domain HRV measurements reviewed; Table S3: Description of main non-linear HRV measurements reviewed.
