# Supplementary material for: Short-Term Heart Rate Variability in Metabolic Syndrome: A Systematic Review and Meta-Analysis
Source: J Clin Med. 2023 Sep 19;12(18):6051. doi: 10.3390/jcm12186051 (PMC10532399; doi:10.3390/jcm12186051)
Supplement: Supplementary file 1 [file jcm-12-06051-s001.zip › jcm-2545235-supplementary.pdf]

Table S1. Description of main time-domain HRV measurements reviewed

PARAMETER	DESCRIPTION
SDNN	Standard deviation of the R-R interval series (overall variability).
rMSSD	The root mean square of differences of successive R-R intervals.
R-R interval	Mean of successive R-R intervals.
pNN50	Successive R-R intervals that differ by more than 50 ms (expressed in percentage)
NN50 (count)	Number of adjacent NN intervals that differ from each other by more than 50 ms.
TINN (ms)	Triangular Interpolation of the NN interval histogram. Baseline width of a histogram displaying NN intervals.
RRtri(ms)	Triangular index. The integral of the sample density distribution of R-R intervals divided by the maximum of the density distribution.

Table S2. Description of main frequency-domain HRV measurements reviewed

PARAMETER	DESCRIPTION
LF, HF, VLF, ULF	Ranges of the spectral components of the HRV<br>HF: high frequency (0.15 – 0.4 Hz)<br>LF: low frequency (0.04 – 0.15 Hz)<br>VLF: very low frequency (0.0033 – 0.04 Hz)<br>ULF: ultra-low frequency (<0.0033 Hz)<br>TP: total power (0.01 – 0.4 Hz)
LF/HF	Ratio between LF and HF bands.

Table S3. Description of main non-linear HRV measurements reviewed

PARAMETER	DESCRIPTION
SD1	Standard deviation of the perpendicular point along the line of identity of the Poincaré plot. It represents the instantaneous beat-to-beat short-term variability.
SD2	Standard deviation of the perpendicular point along the line of identity of the Poincaré plot. It represents the instantaneous beat-to-beat long-term variability.
α1	Short terms fluctuations (4 - 12 beats) of detrended fluctuation analysis. The slopes of a log-log plot (correlation measure as a function of segment length).
ApEn	Approximate entropy, measures the complexity of RR time series (m=2, r=0.2)
ShanEn	Sample entropy, measures the irregularity RR time series (m=2, r=0.2)
